# Multisystem inflammatory syndrome in a neonate with severe hemophilia - a diagnostic challenge in COVID times: a case report

**DOI:** 10.1186/s12887-022-03463-3

**Published:** 2022-07-07

**Authors:** Sumitha Arun, Taliya Grace Cherian, Chepsy Philip

**Affiliations:** 1 Department of Neonatology, Believers Church Medical College Hospital, Thiruvalla, Kerala 689103 India; 2 Department of Hematology, Believers Church Medical College Hospital, Thiruvalla, Kerala 689103 India

**Keywords:** COVID-19, Multisystem inflammatory syndrome newborn (MIS-N), Hemophilia a, Neonatal COVID, Severe hemophilia, Maternal COVID, Intracranial bleed

## Abstract

**Background:**

Multisystem Inflammatory Syndrome in Neonates (MIS-N) can occur following antenatal COVID- 19 infection in the mother. Here we report a rare case of a neonate with Hemophilia A and MIS-N.

**Case presentation:**

A 2-day-old baby presented with an intramuscular hematoma, neonatal seizures, and isolated activated partial thromboplastin time (APTT) prolongation. The neurosonogram showed a subdural hematoma. A diagnosis of Hemophilia A was made and was confirmed by factor 8 assay and genetic analysis. Supportive measures and Factor 8 replacement was initiated. A rising trend of inflammatory markers and an ongoing need for mechanical ventilation were noted. As there was a history of COVID-19 in the mother in the third trimester, MIS-N was diagnosed. The baby was treated with intravenous immunoglobulin (IVIG) and steroids, and there was an improvement in the clinical and laboratory markers. However, the baby developed seizures on day 16. There was an increase in the subdural hemorrhage and a further rise in inflammatory markers. A craniostomy and hematoma evacuation was done and the baby improved.

**Conclusion:**

The concurrent occurrence of hemophilia A with intracranial bleed, and MIS-N in a neonate is a diagnostic challenge. It is important to have a high index of suspicion to ensure timely diagnosis and treatment of MIS-N in this pandemic era.

## Background

Hemophilia A is an X-linked coagulation disorder due to reduced levels of factor 8 with an incidence of 1 in 5000 male childbirth [1].Hemophilia A is confirmed by demonstrating low circulating levels of factor 8 (severe< 1%, moderate 1–5%, mild 5–50% of normal) or by genetic analysis. Severe Hemophilia A may present in the neonatal period.

In this report, we describe the occurrence of Multisystem Inflammatory Syndrome- Neonate (MIS-N) in a 2-day-old baby diagnosed with Hemophilia A. Transplacental transfer of antibodies occurs following maternal COVID-19 disease. This may lead to a hyperinflammatory state in the newborn called Multisystem Inflammatory Syndrome-Neonate (MIS-N). There have been very few cases of MIS-N reported so far [2]. The concomitant occurrence of MIS-N with other disorders, such as Hemophilia A, will pose diagnostic and therapeutic challenges in the pandemic era. This case report highlights the need to have a high index of suspicion to make a timely diagnosis.

### Case presentation

A 2-day-old male baby, born to a primigravida mother at 39 weeks of gestation by a forceps delivery, with a birth weight of 2.8 kg, presented to the Emergency Department (ED) with swelling on the left thigh, poor feeding, and lethargy. There was no family history of bleeding disorder. The baby was noted to have apnea in ED requiring intubation. After admission to the NICU, the baby was ventilated and stabilized. Examination showed bulging anterior fontanelle, anisocoria, and a 3 × 1 cm bluish swelling on the left thigh at the site of vitamin K injection. He had multiple episodes of seizures in the form of tonic posturing of all 4 limbs. Investigations showed anemia (Hemoglobin- 12.8 g/dL), normal leukocyte and platelet counts (Total count- 15,500 cells/μl, platelet-1.6 lac/μl). C-reactive protein (CRP) was 13 mg/L (normal < 10 mg/L). The coagulation profile showed normal prothrombin time (PT) and an isolated activated partial thromboplastin time (APTT) prolongation (PT-20.1 s control-13.3 s, international normalized ratio (INR) -1.60, APTT>120S). A Neurosonogram on day 2 of life, showed a subdural hemorrhage (SDH) of 8 mm in the left frontotemporal region. Packed red cells and fresh frozen plasma were transfused after drawing samples for clotting factors. Factor 8 activity was < 1% and a diagnosis of hemophilia A was made. He was started on Factor 8 at 125 IU twice a day (targeting 100% factor levels), anticonvulsants, and antibiotics (Cefotaxime and Gentamicin). Computerized tomography (CT) scan on day 3 showed an SDH of 10 mm thickness in the left frontotemporoparietal region.

**Fig. 1:**
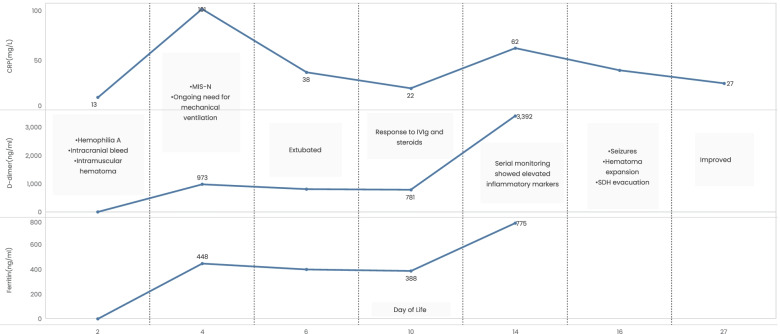
Clinical course and the trend of inflammatory markers in a neonate with Hemophilia A and MIS-N

On day 4 of life, the baby was hemodynamically stable and euglycemic but required continued mechanical ventilation and an increased oxygen requirement. There was also a rising trend in CRP (101 mg/L on day 4) despite changing the antibiotics to Meropenem and Amikacin (Fig. 1). There was conjugated hyperbilirubinemia -total bilirubin of 18.9 mg/dL and direct bilirubin of 2.58 mg/dL.

Considering an antenatal history of maternal SARS-CoV-2 at 32 weeks of gestation, the baby was evaluated for MIS-N. Anti SARS-CoV-2 IgG antibody test was positive (IgG − 4.36- reactive) in the baby. Maternal and neonatal reverse transcription-polymerase chain reaction (RT-PCR) for SARS-CoV-2 were negative. Further investigations revealed elevated inflammatory markers: Lactate dehydrogenase (LDH) -1071 U/L, ferritin-448 ng/ml, and D-dimer-973 ng/ml. Hence the baby was given intravenous immunoglobulin (IVIG - 1 g/kg for 2 days) and methylprednisolone (1 mg/kg every 12 h for 6 days and tapered over 4 days).

The baby improved and the ventilatory requirements were reduced. On day 6, the baby was extubated to room air. The CRP and other inflammatory markers also showed a decreasing trend. An echocardiogram (ECHO) showed prominent left coronaries with aneurysmal dilatation and normal ventricular function (ejection fraction − 77%). Factor 8 was continued targeting 100% levels. Factor 8 inhibitor assay was negative. The intracranial bleed was managed conservatively by the neurosurgery team.

Serial monitoring of laboratory parameters showed an increase in CRP and other inflammatory markers on day 14 (Fig. 1), but the baby continued to improve clinically. However, on day 16 of life, the baby developed seizures and an MRI showed increased bilateral SDH (maximum diameter - 2.3 cm and 0.5 cm on left and right respectively) with a midline-shift to right. There were features suggestive of total brain injury and communicating hydrocephalus. Due to the progression of the intracranial bleed, the baby underwent left parietal craniostomy with subdural hematoma evacuation on day 17 of life. Post-surgery, the seizures were controlled with phenytoin and levetiracetam. The baby was started on breastfeeds. There were no focal neurological deficits. Gene analysis showed a hemizygous intron 22 inversion in the F8 gene on chromosome X (NM_000132.3) confirming Hemophilia A. The baby is now 2-months old and thriving on breast milk alone. He is on factor 8 replacement, is seizure-free on levetiracetam, and is currently under neurodevelopmental follow-up.

## Discussion

Intramuscular hematoma and intracranial bleed (IC bleed) in a male baby with isolated APTT prolongation point to a coagulation disorder. Differential diagnoses to be considered are Factor 8,9,11 and 12 deficiency. In our case, low Factor 8 levels (severe deficiency < 1%) led to the diagnosis of Hemophilia A.

More than one-third of patients with severe hemophilia are diagnosed in the first month of life [3]. They commonly present with a cephalhematoma, an intracranial bleed after an instrumental delivery, or a hematoma at the vitamin K injection site. Therapy is initiated with fresh frozen plasma. Definitive treatment is Factor 8 replacement [4].

In our case, we targeted 100% levels due to intracranial bleed. Despite appropriate treatment, clinical worsening was noted. A thorough investigation for the rare possibility of inhibitors was done and found to be negative. This prompted the treating team to consider an alternate or additional diagnosis. The temporal association of antenatal COVID − 19 infection in the third trimester, the presence of COVID antibodies in the baby, multisystem involvement, and elevated inflammatory markers suggested a diagnosis of MIS-N. Aneurysmal dilatation of proximal coronary arteries in the ECHO provided additional evidence in favor of the diagnosis.

MIS-N is a newly reported entity with few cases reported as yet [5]. The diagnosis is based on Multisystem Inflammatory Syndrome –children (MIS-C) criteria which include clinical and laboratory components [6]. MIS-N can present with cardiac involvement (90%), respiratory failure (40%), and fever (10%) [2]. The treatment modalities include IVIG and steroids, in addition to supportive measures.

This is the first report of MIS-N in a neonate with Hemophilia A to the best of our knowledge. Here we highlight the challenges faced in terms of diagnosis and treatment. Coagulation abnormalities may occur in Multisystem inflammatory syndrome due to COVID-19. Prolonged APTT, elevated fibrinogen, and D – dimer have been reported [7]. However, in this case, the coagulation factor assay showed severe Factor 8 deficiency. This led to the diagnosis of Hemophilia A. Gene analysis showing a pathogenic mutation in the F8 gene in chromosome X confirmed the same. The mutation ruled out the rare possibility of acquired hemophilia from secondary aetiologies including SARS-CoV-2.

Neuronal injury involving perihematomal neurons occurs in IC bleed. This triggers a cascade of events causing the elevation in inflammatory markers. However, there may be elevated inflammatory markers in both MIS-N and resolving or organizing IC bleed [ 8, 9]. There is no single test with high specificity that confirms MIS-N diagnosis. Inflammatory markers including CRP, procalcitonin, D-dimer, ferritin, and N-terminal pro b-type natriuretic peptide (NT-proBNP) are nonspecific. This was a significant concern during treatment.

Aspirin or anticoagulants are recommended for the treatment of babies with dilated coronaries. However, given the ongoing IC bleed, we did not administer aspirin or anticoagulants.

Many issues regarding the diagnosis and management of a neonate with MIS-N may arise especially when it occurs along with other diseases. This case highlights the need for a greater understanding of cases with the concurrent occurrence of MIS-N and other neonatal disorders.

## Conclusion

In this case, we report the occurrence of hemophilia A and COVID-associated MIS-N in a neonate. A high index of suspicion in the current pandemic period may help in diagnosing cases of MIS-N especially when it occurs concurrently with other neonatal disorders.

## Data Availability

Not applicable.

## Abbreviations

MIS-N: Multisystem Inflammatory Syndrome in Neonates
APTT: Activated partial thromboplastin time
IVIG: Intravenous immunoglobulin
ED: Emergency Department
CRP: C-reactive protein
PT: Prothrombin time
INR: International normalized ratio
SDH: Subdural hemorrhage
CT: Computerized tomography
RT-PCR: Reverse transcription- Polymerase chain reaction
LDH: Lactate dehydrogenase
ECHO: Echocardiogram
IC bleed: Intracranial bleed
MIS-C: Multisystem Inflammatory Syndrome –children
NT-proBNP: N-terminal pro b-type natriuretic peptide
